# Identifying a Safety Threshold for Parenteral Glucose Intake in the Early Acute Phase of Preterm Neonates

**DOI:** 10.3390/nu18111821

**Published:** 2026-06-05

**Authors:** Maria Di Chiara, Ilaria Mastropasqua, Flavia Gloria, Arianna Di Domenico, Fabiana Russo, Lucia Dito, Paola Favata, Gianluca Terrin

**Affiliations:** Department of Maternal-Infantile and Urological Sciences, Policlinico Umberto I, Sapienza University of Rome, Viale del Policlinico 155, 00161 Rome, Italyl.dito@policlinicoumberto1.it (L.D.); gianluca.terrin@uniroma1.it (G.T.)

**Keywords:** preterm neonate, parenteral nutrition, dextrose, hyperglycemia, hypertriglyceridemia, EUGR, early acute phase

## Abstract

**Background/Objectives:** The safety of specific parenteral glucose intake values within the range currently recommended by international guidelines for the early acute phase in preterm neonates has not been established. This study aimed to evaluate whether exceeding a data-driven parenteral dextrose intake threshold during the first week of life is independently associated with hyperglycemia, hypertriglyceridemia, metabolic acidosis, and extrauterine growth restriction (EUGR). **Methods:** This was a single-center retrospective study involving preterm neonates (gestational age ≤ 34 weeks and/or birth weight ≤ 1500 g) admitted to the Neonatal Intensive Care Unit of Policlinico Umberto I, Rome, between 2015 and 2022. The analysis followed two pre-specified steps: (1) data-driven identification of an exposure threshold by restricted cubic spline logistic regression; (2) multivariable analyses with the dichotomized exposure, adjusting for gestational age, birth weight, enteral nutrition timing, neonatal morbidity, and perinatal compromise. **Results:** 389 preterm neonates met eligibility. The data-driven inflection point of the spline-derived log-odds curve identified a threshold of 7 g/kg/day. Exceeding this threshold during the first week of life was independently associated with both hyperglycemia (adjusted odds ratio 5.55, 95% confidence interval 2.56 to 12.03; *p* < 0.001) and hypertriglyceridemia (adjusted odds ratio 4.36, 95% confidence interval 1.41 to 13.45; *p* = 0.010), but not with metabolic acidosis or with EUGR at 36 weeks postmenstrual age. The divergence in daily parenteral glucose intake between cases and controls was apparent from the second day of life. **Conclusions:** Exceeding 7 g/kg/day of parenteral dextrose was independently associated with early metabolic complications, but not with growth outcomes. A safety threshold for parenteral glucose may exist within the currently recommended intake range; prospective multicenter studies are needed before clinical recommendations can be drawn.

## 1. Introduction

Preterm neonates, particularly those born very low birth weight (VLBW, <1500 g) or before 34 weeks of gestational age, are critically dependent on parenteral nutrition (PN) during the early postnatal period. The immaturity of the gastrointestinal tract limits the feasibility of full enteral feeding, making intravenous macronutrient supply essential to meet the metabolic demands of this vulnerable population [1,2]. The metabolic trajectory of the preterm neonate is not uniform. Moltu et al., in a position paper jointly endorsed by the leading international scientific societies in paediatric and clinical nutrition, delineated three distinct metabolic phases in the critically ill neonate: early acute, late acute, and recovery [3]. The clinical rationale for providing relatively high macronutrient intakes during the early acute phase is to reduce the risk of extrauterine growth restriction (EUGR), a frequent complication of very preterm infants associated with adverse growth and neurodevelopmental outcomes [4].

During the early acute phase, a period dominated by catabolism and marked metabolic vulnerability, the nutritional support must be carefully calibrated to avoid both under- and over-provision of substrates; However, within the broad intake ranges currently endorsed by international guidelines, both the specific upper boundaries of safe macronutrient delivery and the optimal pace of daily progression toward target intakes during the first week of life remain only partially defined by clinical evidence [3,5].

According to the conceptual framework adopted by international guidelines (Moltu et al., 2021 [3]), the metabolic trajectory of the critically ill neonate is articulated in three sequential phases. The late acute phase, lasting from approximately the second to the seventh day of life depending on individual stabilisation, is characterised by progressive resolution of haemodynamic and endocrine instability and by an increasing tolerance to nutrient escalation. The recovery phase, beginning when clinical stability is achieved, is dominated by anabolism and rapid catch-up growth. The early acute phase, by contrast, is dominated by catabolism, by impaired oxidative capacity for exogenous glucose, and by incompletely suppressible endogenous hepatic glucose production, conditions that together render the neonate uniquely vulnerable to substrate over-provision.

For parenteral glucose supply during this early acute phase, the ESPGHAN/ESPEN/ESPR/CSPEN recommendations propose a broad intake range of 5 to 8 g/kg/day [3]. Glucose is the primary energy substrate for the preterm neonate, and its intravenous administration is initiated promptly after birth. Yet, the metabolic capacity to oxidize parenterally delivered glucose is limited in the first days of life, and exceeding this capacity may lead to significant metabolic complications [6,7]. Hyperglycemia has been consistently associated with increased morbidity and mortality in preterm neonates, including sepsis, intraventricular hemorrhage, retinopathy of prematurity, and bronchopulmonary dysplasia and necrotizing enterocolitis [8,9,10,11,12,13]. Beyond hyperglycemia, excessive parenteral glucose supply may stimulate de novo lipogenesis, contributing to hypertriglyceridemia, and may also trigger metabolic acidosis through increased CO_2_ production from glucose oxidation exceeding the respiratory capacity and lactate accumulation in the setting of anaerobic glycolysis [14,15,16,17].

Despite these well-recognized risks, no study has yet identified a specific parenteral dextrose threshold within the recommended range that discriminates between safe and potentially harmful intakes during the early acute phase.

The aim of the present study was therefore to evaluate whether exceeding a parenteral dextrose intake threshold during the first week of life is independently associated with hyperglycemia, hypertriglyceridemia, and metabolic acidosis, as well as with extrauterine growth restriction (EUGR) as a secondary endpoint, in preterm neonates.

## 2. Materials and Methods

### 2.1. Ethics Approval and Informed Consent

The study was conducted in accordance with the World Medical Association Declaration of Helsinki for research involving human subjects and was approved by the Ethics Committee of Policlinico Umberto I, Sapienza University of Rome (Protocol No. 5089, approved 13 September 2018). In accordance with the institutional policy of our NICU, written informed consent for the use of anonymised clinical data for both clinical care and retrospective observational research is routinely obtained from the parents (or, where applicable, the legally designated guardian) of every neonate at admission. Under Italian and European Union legislation, authorisation by the juvenile court (Giudice Tutelare) is required only when minors are under State guardianship; this circumstance did not apply to any neonate included in the present cohort. 

During the preparation of this manuscript, the authors used Claude Opus 4.7 (Anthropic, San Francisco, CA, USA) solely to assist with English language editing and to improve the readability of selected paragraphs. The tool was not used for study design, data collection, statistical analysis, generation or interpretation of scientific content, or production of figures and tables. All AI-assisted outputs were critically reviewed, verified and edited by the authors, who take full responsibility for the integrity, accuracy and originality of the content of this publication.

### 2.2. Study Design and Population

This was a single-centre retrospective study. The study was conducted at the Neonatal Intensive Care Unit (NICU) of Policlinico Umberto I, Sapienza University of Rome (Rome, Italy). Clinical, nutritional, laboratory and outcome data had been prospectively recorded during routine clinical care, contemporaneously with each event, on a standardised research database maintained by dedicated NICU staff.

The study cohort included all neonates consecutively admitted to the NICU between 1 January 2015 and 31 December 2022 with a gestational age at birth ≤ 34 completed weeks and/or a birth weight ≤ 1500 g, and who had received parenteral nutrition (PN) for at least the first seven consecutive days of life. Neonates were excluded if they had a major congenital malformation, an inborn error of metabolism, a congenital infection, a congenital or acquired immunodeficiency, a chromosomal abnormality, loss of central venous access during the first week of life, transfer to another centre within the first 72 h of life, or death within the first 72 h of life.

The early acute phase was defined operationally as the first eight calendar days of life, i.e., day 0 (day of birth) through day 7 inclusive [3].

The exposure of interest was the parenteral dextrose intake actually received during the early acute phase, expressed in grams per kilogram of body weight per day (g/kg/day). For each neonate, the daily dextrose intake actually received was computed from the prospectively recorded values of the prescribed dextrose concentration, the total volume of parenteral solution effectively administered over each 24-h period, and the body weight on the corresponding day of life. This metric was preferred over prescribed intake because it reflects the actual glucose load delivered to the infant and accounts for any discrepancy between prescribed and administered volumes.

The analytical strategy was structured in two sequential, pre-specified steps. In the first step, an exposure threshold associated with an increased probability of metabolic complications was identified data-driven by means of a dose–response analysis of the peak daily parenteral dextrose intake observed during the early acute phase (see Section 2.6). In the second step, the threshold derived in step 1 was used to dichotomise the exposure (peak intake above the threshold on at least one day during the first week of life vs. at or below the threshold on every day), and the dichotomised exposure was carried into all subsequent univariate and multivariable analyses.

### 2.3. Outcome Definitions

The primary outcomes were the occurrence, during the first week of life, of hyperglycaemia, defined as two consecutive blood glucose measurements above 180 mg/dL obtained at an interval of at least three hours, in accordance with Galderisi et al. [18]; hypertriglyceridaemia, defined as at least one serum triglyceride measurement above 150 mg/dL, in accordance with Chan [19]; and metabolic acidosis, defined as an arterial or capillary pH < 7.25 with a base excess < −6 mmol/L, in accordance with the criteria adopted for preterm neonates in the Cochrane systematic review by Keir et al. [20].

The pre-specified secondary outcome was extrauterine growth restriction (EUGR), defined longitudinally, consistent with the definition previously adopted by our group, as a decline in weight Z-score greater than 1.0 between birth and the postmenstrual age of 36 weeks (or hospital discharge, whichever occurred first), with Z-scores computed according to the Fenton growth reference [21,22]. Growth velocity at 36 weeks postmenstrual age, computed from regaining birth weight to 36 weeks PMA using the exponential method described, was analysed as a continuous exploratory outcome supporting the EUGR analysis [23].

### 2.4. Nutritional Protocol

The clinical and nutritional practices described in this section reflect the standard of care of the participating NICU during the entire study period (2015–2022). In our NICU, individualized PN was initiated within 24 h after admission in all enrolled neonates. PN prescriptions were formulated daily by the attending neonatologist based on each infant’s clinical condition, laboratory findings, and body weight, and were compounded by the hospital pharmacy using dedicated prescription software. PN was administered through central venous access.

Total fluid intake from combined PN and enteral nutrition (EN) was initiated at 70–90 mL/kg/day and progressively increased by 10–20 mL/kg/day until reaching 150–180 mL/kg/day. The parenteral macronutrient requirements were calculated by subtracting the enteral nutrition intake from the estimated total nutritional requirement. Glucose was administered as dextrose in individualized concentrations in order to maintain blood glucose levels within the target range of 50–180 mg/dL. Parenteral nutrition was complete and included, in addition to glucose, parenteral amino acids (typically initiated at approximately 1.0 g/kg/day on the first day of life and progressively advanced to approximately 2.5 g/kg/day by the end of the first week) and intravenous lipid emulsion (typically initiated at approximately 0.3 g/kg/day and progressively advanced to approximately 1.8 g/kg/day), together with electrolytes (sodium, potassium, calcium, magnesium and phosphorus), in accordance with standard ESPGHAN recommendations for the early acute phase.

The stability of the effective intraday glucose infusion rate was ensured by the local NICU protocol. Catch-up infusions to recover transiently interrupted parenteral nutrition were not allowed. Whenever an interruption of parenteral nutrition longer than 10 min was required to administer a medication, a 10% dextrose infusion was simultaneously started through a separate venous access at the same glucose rate as the ongoing parenteral nutrition, so that the effective rate of glucose delivery to the infant was maintained without negative dips or compensatory peaks. Vasoactive drugs (dopamine, dobutamine, adrenaline) and other parenteral medications were diluted in 5% dextrose at low volumes, contributing a clinically negligible additional glucose load.

Blood glucose monitoring during the first week of life followed a protocol-driven schedule, uniform across the cohort. During the first 72 h of life, capillary or arterial blood glucose was measured every 6 h in all neonates receiving parenteral nutrition; whenever a value exceeded 180 mg/dL, the measurement was repeated 3 h later, and hyperglycaemia was confirmed only if the second measurement also exceeded 180 mg/dL. After the first 72 h, blood glucose was measured every 12 h.

In case of confirmed hyperglycaemia, the parenteral glucose intake was reduced according to ESPGHAN recommendations; insulin infusion was reserved as a second-line strategy when blood glucose remained above 180 mg/dL despite glucose reduction. In the present cohort, 38 of 371 neonates (10.2%) received insulin during the first week of life.

The enteral feeding protocol was uniform throughout the study period. Minimal enteral feeding (MEF) was initiated within 48 h after birth at 10–20 mL/kg/day and advanced by 20–30 mL/kg/day if tolerated. Enteral feeding was withheld in the presence of severe abdominal distension, emesis, ileus with visible bowel loops, blood in stools, apnea, bradycardia, signs of inadequate perfusion, or hemodynamic instability. Fresh, unfortified human milk from the infant’s own mother was administered as soon as available; preterm formula was used when human milk was unavailable or insufficient.

### 2.5. Data Collection

For each neonate, demographic, perinatal, postnatal, nutritional, laboratory and outcome data had been prospectively recorded contemporaneously with each event on a standardised research database maintained by dedicated NICU staff, using the same data-collection protocol and the same operational definitions previously described by our group [21]. Variables collected encompassed maternal and obstetric characteristics, perinatal indicators of compromise, postnatal anthropometrics, daily parenteral and enteral macronutrient intakes during the first week of life, routine laboratory monitoring, and prematurity-related morbidities.

For the exposure of interest specific to the present analysis, the daily parenteral dextrose intake actually received was recorded as a continuous variable, in g/kg/day, from day 0 through day 7 inclusive. The dichotomised exposure used in the second analytical step was derived from this continuous variable at the analysis stage, using the threshold identified in the dose–response analysis.

### 2.6. Statistical Analysis

Descriptive and bivariate analyses were performed with SPSS version 27.0 (IBM Corp., Chicago, IL, USA); restricted cubic spline regression and the multivariable models were fitted with R version 4.3 (R Foundation for Statistical Computing) using the rms package version 6.8-1 [24]. The normality of continuous variables was assessed with the Shapiro–Wilk test; continuous variables were summarised as mean ± standard deviation or median (interquartile range), depending on distribution, and compared with the Student *t* test or the Mann–Whitney U test. Categorical variables were summarised as frequencies (percentages) and compared with the chi-square or Fisher exact test. All tests were two-sided; *p* < 0.05 was considered significant. No imputation procedure was applied to missing data; complete-case analysis was performed for each multivariable model.

The analysis followed two pre-specified sequential steps.

In the first step, the dose–response relationship between the peak daily parenteral dextrose intake during the first week of life and the probability of each primary metabolic outcome (hyperglycaemia, hypertriglyceridaemia) was modelled with restricted cubic spline logistic regression, with four knots placed at the 5th, 35th, 65th and 95th percentiles of the exposure distribution. The overall association was tested with a Wald chi-square statistic on all spline coefficients jointly; non-linearity was tested with a Wald chi-square on the spline coefficients beyond the linear basis. The data-driven inflection point of the spline-derived log-odds curve was adopted as the operational threshold and used as the reference value for scaling the dose–response curves.

In the second step, the exposure was dichotomised at the threshold derived in step 1 (peak intake above the threshold on at least one day during the first week of life vs. always at or below). For each primary outcome (hyperglycaemia, hypertriglyceridaemia, metabolic acidosis), for the pre-specified secondary outcome of extrauterine growth restriction (EUGR), and for the exploratory outcome of growth velocity at 36 weeks postmenstrual age, a univariate comparison between exposed and unexposed neonates was performed. According to the pre-specified plan, a binary logistic regression multivariable model was fitted for those primary outcomes showing a statistically significant univariate association with the dichotomised exposure.

All multivariable models adjusted simultaneously for the dichotomised exposure and for clinically relevant confounders pre-specified for each outcome (gestational age < 32 weeks, ELBW, delayed enteral nutrition, neonatal morbidity, perinatal compromise. Effect estimates were reported as adjusted odds ratios with 95% Wald confidence intervals. The composite covariate “neonatal morbidity” was operationally defined as the occurrence of culture-proven sepsis and/or intraventricular haemorrhage of any grade.

Before fitting the multivariable models, collinearity among the candidate covariates was assessed by computing the variance inflation factor for each covariate, the phi coefficient between ELBW and gestational age < 32 weeks, and the condition number of the design matrix; all diagnostics indicated absence of problematic collinearity (variance inflation factor ≤ 1.40 for both binary descriptors of preterm vulnerability, phi coefficient ≤ 0.232, condition number of the design matrix < 7), supporting their simultaneous inclusion in the multivariable models.

To assess the internal stability of the multivariable models for the two primary metabolic outcomes (hyperglycaemia and hypertriglyceridaemia), two pre-specified internal validation procedures were performed on the analytic cohort. First, non-parametric bootstrap resampling with 1000 resamples drawn with replacement and stratified by outcome was used to compute bias-corrected and accelerated (BCa) 95% confidence intervals for each adjusted odds ratio, with the acceleration parameter estimated by leave-one-out jackknife. Second, stratified 10-fold cross-validation was repeated 100 times with random fold assignment to obtain the apparent and cross-validated Harrell C statistic, the corresponding optimism (apparent minus cross-validated), and the cross-validated calibration slope of each multivariable model. A fixed random seed was used to ensure reproducibility. Analyses were performed in Python 3.11.7 with the statsmodels and scikit-learn libraries.

## 3. Results

### 3.1. Study Population

A total of 528 neonates were consecutively admitted to the NICU during the study period. After applying the inclusion and exclusion criteria, 389 preterm neonates met eligibility and constituted the analytic cohort.

### 3.2. Baseline Characteristics

Baseline maternal, perinatal, and postnatal characteristics of the analytic cohort are reported in Table 1.

### 3.3. Primary Outcomes

#### 3.3.1. Identification of the Exposure Threshold

**Figure 1:**
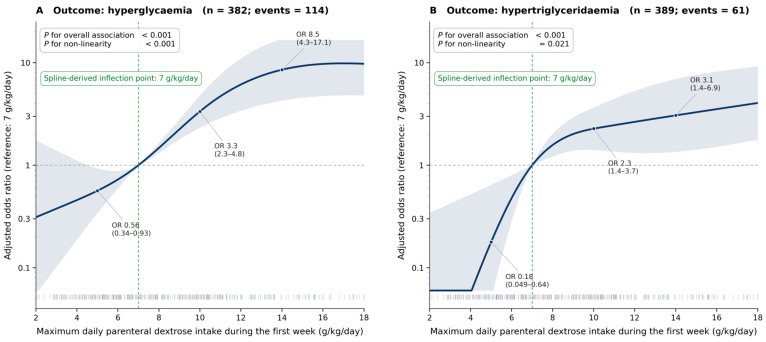
Restricted cubic spline dose–response between peak parenteral dextrose intake during the first week of life and early metabolic complications. Adjusted odds ratios (OR) for the binary outcomes are plotted as a continuous function of peak parenteral dextrose intake during the first week of life (g/kg/day), modelled by restricted cubic splines with four knots placed at the Harrell percentiles 5%, 35%, 65% and 95% of the exposure distribution. The reference value used to scale OR was 7 g/kg/day, which corresponds to the data-driven inflection point identified by the spline (knots, panel (**A**): 3.35, 6.72, 10.46, 19.49 g/kg/day; panel (**B**): 3.35, 6.70, 10.46, 19.40 g/kg/day). The solid blue curve is the point estimate; the shaded band is the pointwise 95% confidence interval. The vertical green dashed line marks the spline-derived inflection point (7 g/kg/day); the horizontal grey dashed line marks OR = 1 (no association). Annotations along each curve report OR with 95% CI at selected exposure values. The rug at the foot of each panel shows the observed peak intake values in the analysed cohort. Panel (**A**): outcome, hyperglycaemia in the first week of life (*n* = 382; events = 114). Panel (**B**): outcome, hypertriglyceridaemia in the first week of life (*n* = 389; events = 61). Both metabolic outcomes were defined as the occurrence of at least one episode meeting the local NICU laboratory criteria during postnatal days 0 to 7. The exposure was defined as the maximum daily dose of parenteral dextrose actually received between postnatal day 0 and day 7. Reported *p*-values are from Wald tests: *p* for overall association tests the joint null hypothesis that all spline coefficients are zero (i.e., no exposure effect); *p* for non-linearity tests the joint null hypothesis that the spline coefficients beyond the linear basis are zero (i.e., a strictly linear log-OR vs. intake relationship). The 7 g/kg/day inflection point was identified in-sample from the same cohort used in subsequent multivariable analyses; the corresponding effect estimates may be subject to optimism bias and require external validation.

In the dose–response analysis, peak parenteral dextrose intake during the first week of life showed a non-linear association with the probability of hyperglycaemia and hypertriglyceridaemia. The data-driven inflection point of the spline-derived log-odds curve was 7 g/kg/day; the dose–response curves with 95% confidence bands and the *p*-values for overall association and non-linearity are shown in Figure 1. The cumulative parenteral dextrose intake actually received during the first week of life was, on average, 48.08 ± 28.77 g/kg (median 43.90, interquartile range 25.34 to 63.52, range 1.52 to 147.14 g/kg).

#### 3.3.2. Incidence and Univariate Analyses

**Figure 2:**
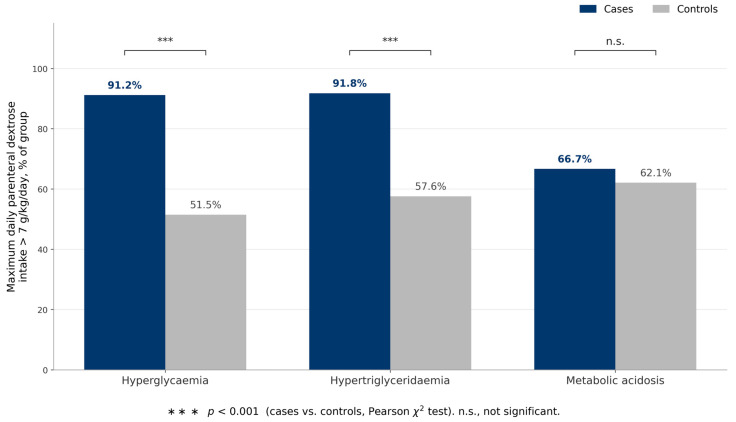
Proportion of preterm neonates whose peak parenteral dextrose intake exceeded the threshold of 7 g/kg/day during the first week of life, stratified by primary metabolic outcome (cases vs. controls). The bar chart shows the percentage of neonates whose peak parenteral dextrose intake exceeded 7 g/kg/day on at least one day during the first week of life, separately for cases (neonates who developed the metabolic complication) and controls (neonates who did not), for each primary outcome: hyperglycaemia, hypertriglyceridaemia, and metabolic acidosis. Asterisks denote statistically significant differences between cases and controls (chi-square test, *p* < 0.05). Definitions: hyperglycaemia, two consecutive blood glucose measurements above 180 mg/dL obtained at an interval of at least three hours [18]; hypertriglyceridaemia, at least one serum triglyceride measurement above 150 mg/dL [19]; metabolic acidosis was defined as an arterial or capillary pH < 7.25 with a base excess < −6 mmol/L, in accordance with the operational criteria adopted in observational studies of preterm neonates [25]. The 7 g/kg/day threshold corresponds to the data-driven inflection point identified in the restricted cubic spline dose–response analysis (Figure 1). *n* = 389 preterm neonates.

Hyperglycaemia, hypertriglyceridaemia, and metabolic acidosis were observed in 114 (29.3%), 61 (15.7%), and 147 (37.8%) of the 389 neonates of the analytic cohort, respectively. The proportions of neonates whose peak parenteral dextrose intake exceeded 7 g/kg/day on at least one day during the first week of life, stratified by metabolic outcome (cases, i.e., neonates who developed the metabolic complication, vs. controls, i.e., neonates who did not), are shown in Figure 2. Exceeding the threshold was statistically significantly more frequent among cases of hyperglycaemia and hypertriglyceridaemia than among controls; no statistically significant difference was observed for metabolic acidosis.

#### 3.3.3. Multivariable Analyses

**Figure 3:**
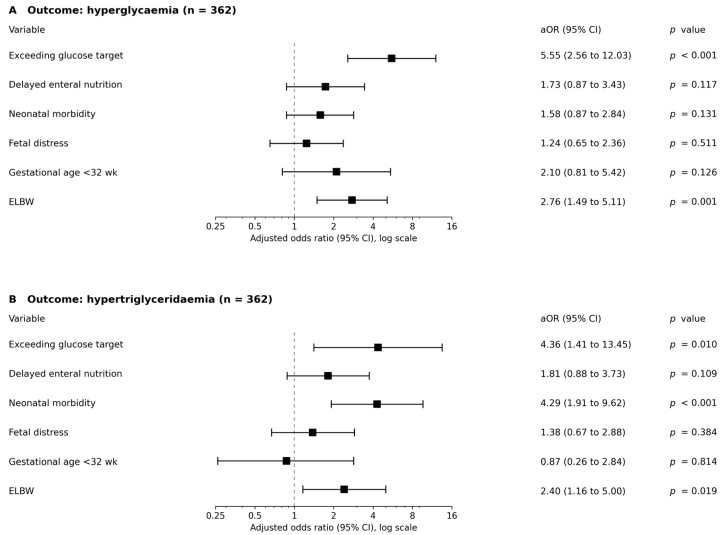
Multivariable models for early-week metabolic complications. Forest plots show adjusted odds ratios (aOR) with 95% confidence intervals (CI) from two multivariable logistic regression models, one for each outcome. Each model was simultaneously adjusted for all six variables listed in the panel. The *x*-axis is plotted on a logarithmic scale; the dashed vertical line marks OR = 1 (no association). Filled squares denote point estimates; horizontal whiskers denote 95% CI. Reported *p*-values are from Wald tests; no adjustment for multiple comparisons was applied. Panel (**A**): outcome, hyperglycaemia in the first week of life (*n* = 362 neonates with complete covariate data). Panel (**B**): outcome, hypertriglyceridaemia in the first week of life (*n* = 362). Variables: Exceeding glucose target, peak parenteral dextrose intake > 7 g/kg/day during the first week of life (data-driven inflection point from a preceding restricted cubic-spline analysis); delayed enteral nutrition, enteral feeding initiated after 72 h of life; neonatal morbidity, culture-proven sepsis and/or intraventricular haemorrhage (IVH) of any grade; fetal distress, 5-min APGAR score < 5 and/or umbilical cord pH < 7.2; gestational age < 32 weeks, dichotomous; ELBW, extremely low birth weight (<1000 g). The 95% confidence intervals plotted in the figure are the apparent Wald intervals from the multivariable logistic regression on the analytic cohort. Bias-corrected and accelerated 95% confidence intervals obtained by non-parametric bootstrap resampling with 1000 stratified resamples are reported in Supplementary Table S1.

In accordance with the pre-specified analytical plan, a multivariable logistic regression model was fitted for hyperglycaemia and hypertriglyceridaemia (significant univariate association), and not for metabolic acidosis. After adjustment for the pre-specified covariates, exceeding the 7 g/kg/day threshold during the first week of life remained independently associated with both hyperglycaemia and hypertriglyceridaemia. Adjusted odds ratios, 95% confidence intervals, and Wald *p*-values for all covariates of both models are reported in Figure 3.

#### 3.3.4. Internal Validation

Internal validation of the multivariable models for hyperglycaemia and hypertriglyceridaemia by non-parametric bootstrap resampling and by stratified 10-fold cross-validation indicated stable estimates of the exposure-outcome association. The bias-corrected and accelerated 95% confidence intervals for the adjusted odds ratio of exceeding the 7 g/kg/day threshold excluded the null in both models; the optimism in discrimination was modest, and the cross-validated calibration slopes were slightly below unity, compatible with a quantitatively contained overoptimism of the predicted probabilities. Detailed estimates for every covariate are reported in Supplementary Table S1; the apparent and cross-validated discrimination and calibration metrics are reported in Supplementary Table S2.

### 3.4. Secondary Outcome: Growth at 36 Weeks Postmenstrual Age

**Figure 4:**
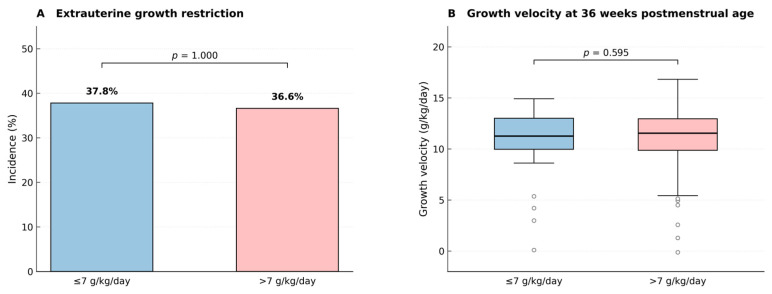
Growth outcomes at 36 weeks postmenstrual age in preterm neonates stratified by parenteral dextrose intake exceeding the threshold during the first week of life. (**A**) Bar chart showing the incidence of extrauterine growth restriction (EUGR) at 36 weeks postmenstrual age in neonates whose peak parenteral dextrose intake during the first week of life remained at or below the threshold (≤7 g/kg/day) and in those whose peak intake exceeded the threshold (>7 g/kg/day). Numerical values above each bar indicate the proportion of EUGR within each group; the *p*-value derives from the Fisher exact test. (**B**) Box plot of growth velocity (g/kg/day) at 36 weeks postmenstrual age in the same two groups. Boxes represent the interquartile range, horizontal lines within the boxes represent the median, whiskers extend to 1.5 × the interquartile range, and open circles represent values beyond the whiskers; the *p*-value derives from the Mann–Whitney U test. Abbreviations: EUGR, extrauterine growth restriction; PMA, postmenstrual age.

EUGR was ascertained in 189 neonates of the analytic cohort, and the exploratory continuous outcome of growth velocity at 36 weeks postmenstrual age was ascertainable in 174 neonates. At univariate analysis, neither the proportion of neonates meeting the EUGR definition (*p* = 1.000) nor mean growth velocity (*p* = 0.595) differed between exposed and unexposed groups; both univariate distributions are shown in Figure 4.

### 3.5. Additional Analyses

Daily parenteral dextrose intake during the first week of life, stratified by primary metabolic outcome (cases vs. controls) for hyperglycaemia and hypertriglyceridaemia, is shown in Supplementary Figure S1.

## 4. Discussion

### 4.1. Summary of Principal Findings

The present study shows that exceeding a parenteral dextrose intake threshold during the first week of life is independently associated with the development of hyperglycemia and hypertriglyceridemia in preterm neonates. This association persisted after adjustment for gestational age, birth weight, delayed enteral nutrition, neonatal morbidity, and perinatal compromise, indicating that parenteral glucose exposure represents a distinct and potentially modifiable risk factor beyond the well-established contribution of immaturity and clinical severity. The threshold identified in the population we examined lies within the upper portion of the range currently recommended by international guidelines for the early acute phase of critical illness in preterm neonates [3], and to our knowledge no previous study has specifically evaluated a parenteral glucose intake threshold within the recommended range as an independent predictor of metabolic complications in preterm neonates.

### 4.2. Comparison with Existing Literature: Hyperglycemia

In a previous randomized controlled trial conducted at our institution, we demonstrated that early achievement of macronutrient targets in parenteral nutrition significantly increased the incidence of hyperglycemia compared to a more gradual approach in VLBW neonates [21]. That study provided the first controlled evidence that the rate of nutritional escalation during the first week of life directly affects glycemic outcomes in this population. The present study extends those findings by identifying a specific parenteral glucose intake threshold (7 g/kg/day) as an independent predictor of hyperglycemia, offering a quantitative target that complements our previous observation on the pace of macronutrient advancement.

Notably, the day-by-day analysis of parenteral glucose intake in our cohort provides a temporal dimension to these findings. The divergence in glucose intake between neonates who developed metabolic complications and those who did not was already apparent by the second day of life. Neonates who remained free of hyperglycemia and hypertriglyceridemia followed a more gradual escalation trajectory, reaching the threshold of 7 g/kg/day only in the second half of the first week, whereas those who developed complications exceeded this value as early as day 2 and continued to diverge progressively thereafter. This pattern suggests that the pace of glucose escalation in the first days of the acute phase may be as clinically relevant as the absolute daily intake. Current international guidelines define the early acute phase and recommend a glucose intake range for this period, but do not provide specific guidance on the optimal day-by-day progression within that range [3]. Our data suggest that a more gradual trajectory, allowing glucose intake to approach the upper portion of the recommended range only after the first few days of metabolic stabilization, may characterize a lower-risk nutritional course.

Stensvold et al., in a large prospective cohort of extremely preterm neonates, reported that enhanced parenteral nutrition was associated with an increased risk of hyperglycemia, and that hyperglycemia itself was an independent risk factor for mortality [9]. Although their study did not identify a specific glucose threshold, their findings are consistent with our observation that higher parenteral glucose delivery during the first week of life increases the risk of metabolic complications. Zamir et al. confirmed the association between higher caloric intake via parenteral nutrition and hyperglycemia in extremely preterm infants [26], while Tottman et al. demonstrated that higher macronutrient intakes were associated with increased metabolic instability in very preterm neonates, including hyperglycemia requiring insulin therapy [27].

Not all studies, however, have reached the same conclusion. Beardsall et al., in the NIRTURE cohort, reported no association between glucose infusion rate and hyperglycemia in very low birth weight infants, identifying prematurity, small size, and sepsis as the principal determinants [28]. These apparently discrepant findings may be partly explained by differences in how glucose exposure was measured across studies. Most previous investigations, including those by Stensvold and Beardsall, quantified glucose delivery as an infusion rate (expressed in mg/kg/min) captured at a specific time point or as a daily maximum. This metric reflects the instantaneous speed of glucose administration but does not account for variations in infusion rate occurring throughout the day. In our study, glucose exposure was defined differently: for each day of the first week of life, we calculated the total amount of parenteral glucose actually received over 24 h, expressed in g/kg/day. A neonate was classified as exposed if this daily total exceeded 7 g/kg/day on at least one day. This metric reflects the actual glucose load delivered over an entire day, regardless of how the infusion rate varied during that period, and may therefore provide a more clinically meaningful measure of glucose exposure during the escalation phase of parenteral nutrition. This choice is further supported by three converging considerations: international guidelines for the early acute phase express the recommended boundaries in g/kg/day rather than in instantaneous infusion rate, so the exposure metric is aligned with the metric on which the prescribing clinician acts; the pathophysiological mechanism we are testing, namely saturation of the limited glucose oxidative capacity, is a function of cumulative load over 24 h rather than of an instantaneous rate; and the convergence of cohort studies using cumulative intake metrics in detecting an association with hyperglycaemia, in contrast with studies based on a single time-point infusion rate, is consistent with this interpretation.

### 4.3. Hypertriglyceridemia and Metabolic Acidosis

The association between parenteral glucose intake and hypertriglyceridemia in preterm neonates has received far less attention in the literature. Previous studies on hypertriglyceridemia in this population have focused predominantly on intravenous lipid emulsions as the primary nutritional determinant. To our knowledge, no prior study has specifically evaluated parenteral glucose intake as an independent predictor of hypertriglyceridemia. Our finding that exceeding 7 g/kg/day of parenteral glucose was independently associated with hypertriglyceridemia, even after adjusting for birth weight, gestational age, and neonatal morbidity, suggests that excessive glucose delivery may contribute to triglyceride elevation through mechanisms beyond lipid administration alone, a hypothesis supported by the known role of glucose as a substrate for hepatic de novo lipogenesis [14].

Notably, neonatal morbidity emerged as the strongest independent predictor of hypertriglyceridemia in our model, while it did not reach significance for hyperglycemia. This divergence suggests that the pathophysiology underlying these two complications may differ: hyperglycemia appears to be more directly driven by substrate overload, whereas hypertriglyceridemia may require the concurrent presence of systemic inflammation to fully manifest.

Exceeding the parenteral glucose threshold was not significantly associated with metabolic acidosis. This finding is not unexpected, as metabolic acidosis in preterm neonates is recognized to have a multifactorial etiology, including renal tubular immaturity, hemodynamic instability, and sepsis, in which the contribution of glucose excess is likely marginal compared to other determinants.

### 4.4. Growth Outcome and the Clinical Relevance of the Threshold

In our cohort, exceeding the parenteral dextrose threshold during the first week of life was not significantly associated with EUGR at univariate analysis. This observation carries a clinical relevance that extends beyond the metabolic outcomes themselves. The rationale for delivering parenteral glucose intakes in the upper portion of the recommended range during the early acute phase rests primarily on the prevention of the cumulative energy deficit and the consequent EUGR which together represent the principal nutritional concern in the care of the very preterm neonate [4,29,30]. In our population, however, intakes above the identified threshold did not translate into a detectable advantage on growth outcome, while being independently associated with hyperglycemia and hypertriglyceridemia. Taken together, the absence of a measurable nutritional benefit on growth and the documented increase in the risk of metabolic complications associated with intakes above the threshold reinforce the clinical relevance of the threshold itself. From the perspective of the neonatologist, this finding shifts the risk–benefit balance against intakes exceeding the threshold during the early acute phase, since the principal clinical objective justifying high parenteral glucose delivery, namely the prevention of postnatal growth restriction, is not supported by our data. Beyond growth, evidence from our group has previously shown that, in preterm newborns, higher parenteral, as opposed to enteral, nutritional intake in the first days of life is associated with lower serum levels of nerve growth factor (NGF) and brain-derived neurotrophic factor (BDNF) at 28 days of postnatal life, suggesting that the choice of nutritional strategy in this population may have implications that extend beyond growth and metabolic outcomes [31].

### 4.5. Pathophysiological Considerations

The first week of life in preterm neonates corresponds to what international guidelines define as the early acute phase of critical illness, a period characterized by hemodynamic instability, endocrine immaturity, and an active stress response during the transition to extrauterine life [3,32]. During this phase, the metabolic capacity of the preterm neonate is profoundly limited. The ability to oxidize exogenous glucose is reduced, peripheral insulin sensitivity is impaired, and, critically, hepatic endogenous glucose production is not fully suppressible by exogenous glucose infusion [6,7]. This means that parenteral glucose does not simply replace endogenous production but is, in part, added to it, creating a cumulative glucose load that exceeds the oxidative capacity of the neonate. It is precisely in this phase of metabolic vulnerability that parenteral nutrition is initiated and rapidly escalated, and it is in this same window that our study demonstrates the association between exceeding 7 g/kg/day of parenteral dextrose and the development of hyperglycemia.

The concept that the early acute phase requires a different nutritional approach compared to the subsequent stable and growth phases has been increasingly recognized [5]. Current guidelines acknowledge that metabolic tolerance during the first days of life is lower than in later periods, and that the nutritional strategy should be modulated according to the clinical condition of the neonate rather than following a uniform escalation protocol [3]. Our findings support this principle by demonstrating that a glucose intake within the upper portion of the recommended range for the acute phase is already independently associated with metabolic complications, suggesting that the margin of safety during this period may be narrower than currently assumed.

Once established, hyperglycemia itself perpetuates a cycle of metabolic harm. As previously demonstrated by our group, hyperglycemia induces a proinflammatory cytokine response and increases oxidative stress through caspase activation and reactive oxygen species production in preterm neonates, mechanisms that contribute to the pathogenesis of major neonatal morbidities [8,21,33]. This concept of glucotoxicity implies that the damage extends beyond the initial substrate overload into a self-sustaining cascade of inflammatory and oxidative injury, further compromising the already limited metabolic reserve of the critically ill preterm neonate.

The independent association between parenteral glucose excess and hypertriglyceridemia observed in our study is consistent with the known role of glucose as the primary substrate for hepatic de novo lipogenesis [14]. When glucose supply exceeds oxidative needs during the acute phase, the surplus is channeled toward fatty acid synthesis in the liver, with subsequent export as triglyceride-rich lipoproteins. In the preterm neonate, the limited capacity for peripheral lipoprotein clearance may further amplify this effect, resulting in elevated circulating triglyceride levels even in the absence of excessive lipid administration [15]. The observation that neonatal morbidity was the strongest independent predictor of hypertriglyceridemia, while it did not reach significance for hyperglycemia, suggests that systemic inflammation may impair lipoprotein lipase activity, creating a synergistic mechanism between glucose-driven lipogenesis and inflammation-mediated clearance impairment [34]. This divergence reinforces the idea that the pathophysiology of these two complications, although sharing a common nutritional trigger, involves distinct metabolic pathways that are differently modulated by the clinical severity of the acute phase.

### 4.6. Implications for Current Nutritional Guidelines

The parenteral glucose threshold identified in this study lies within the range currently recommended by international guidelines for the early acute phase of parenteral nutrition in preterm neonates. The most recent ESPGHAN position paper proposes a glucose intake range for the initial phase of critical illness, while acknowledging that individual tolerance may vary considerably [3]. Earlier guidelines proposed an even broader range, without distinguishing between clinical phases [32]. Our observation that exceeding the identified threshold is independently associated with the development of hyperglycemia and hypertriglyceridemia does not contradict the existing recommendations, but rather suggests that the margin between adequate glucose provision and metabolic overload during the acute phase may be narrower than the breadth of the recommended range implies.

Current guidelines are largely based on expert consensus and on a limited number of controlled trials conducted in heterogeneous preterm populations, as acknowledged by the guideline authors themselves [3]. The evidence supporting specific glucose intake targets during the first days of life remains sparse, particularly for the most vulnerable subgroups such as extremely low birth weight neonates, who in our cohort showed a higher risk of both hyperglycemia and hypertriglyceridemia even after adjustment for glucose intake. This observation suggests that the recommended range, while appropriate as a general framework, may require a more individualized interpretation when applied to neonates in the early acute phase, in which metabolic tolerance is most constrained.

Our data do not allow us to propose a definitive alternative threshold, nor do they imply that intakes below the identified threshold eliminate the risk of metabolic complications. They do, however, suggest that within the currently recommended range there may exist a gradient of risk, and that a cautious approach to glucose escalation during the first week of life, particularly in the most immature neonates, may be warranted. This perspective is consistent with the evolving concept, endorsed by recent guidelines, that nutritional support during critical illness should be tailored to the clinical phase and to the individual metabolic capacity of the patient, rather than following a uniform escalation protocol [3,5].

The dose–response analysis performed by means of restricted cubic splines characterized the relationship between parenteral glucose intake during the first week of life and the probability of metabolic complications, identifying a threshold above which the risk of hyperglycemia and hypertriglyceridemia appeared to increase progressively. Below this threshold, the probability of these outcomes remained low and relatively stable, whereas above it the risk increased with the magnitude of glucose intake. The significant non-linearity of this relationship suggests that a simple linear model would not adequately capture the dose–response pattern and is consistent with the existence of a threshold effect at which the metabolic tolerance of the preterm neonate may be exceeded. This observation is biologically plausible, as it is consistent with the saturation of the oxidative capacity for exogenous glucose described in the pathophysiological literature [6,7]. These findings, however, derive from a single-center retrospective cohort, and the threshold identified in our analysis should be interpreted as an exploratory data-driven estimate that requires confirmation in independent populations, and ideally in prospective multicenter studies, before being translated into clinical practice or used to inform a revision of the current intake ranges.

### 4.7. A Potentially Modifiable Risk Factor

Among the independent predictors of metabolic complications identified in our study, parenteral glucose intake is the only factor that is directly amenable to clinical intervention. Extremely low birth weight and gestational age are inherent characteristics of the patient that cannot be modified, and neonatal morbidity, while potentially influenced by overall care, is largely determined by the severity of the clinical course. In contrast, the daily amount of parenteral glucose delivered is a prescriptive decision made by the clinician and can be adjusted on a day-by-day basis according to the metabolic response of the individual neonate.

This distinction carries practical implications. Our day-by-day analysis of glucose intake during the first week of life provides a temporal characterization of when the threshold of 7 g/kg/day is most frequently exceeded, offering a concrete time window during which closer monitoring of glucose prescription and metabolic response may be most relevant. The identification of a specific, quantifiable nutritional target, rather than a general recommendation to limit glucose escalation, may facilitate the translation of these findings into daily clinical practice in the neonatal intensive care unit. While our observational design does not allow us to conclude that reducing parenteral glucose intake below this threshold would prevent metabolic complications, the independent and consistent nature of the association across two distinct outcomes suggests that this variable deserves consideration as a modifiable target in nutritional protocols for the early acute phase.

### 4.8. Strengths and Limitations

This study has several strengths. The sample size of 362 neonates with complete data for all variables included in the multivariate analysis is relatively large for a single-center study in this population. The use of binary logistic regression with adjustment for clinically relevant confounders, including birth weight, gestational age, delayed enteral nutrition, neonatal morbidity, and perinatal compromise, allowed us to isolate the independent contribution of parenteral glucose intake from the well-known effects of immaturity and clinical severity. The definition of glucose exposure was based on the actual amount of parenteral glucose received over each 24-h period, calculated from the prescribed glucose concentration and the volume effectively administered, rather than on a single time-point measurement of infusion rate. This approach provides a more comprehensive assessment of the daily glucose load than metrics based on instantaneous infusion rates. Furthermore, the day-by-day evaluation of glucose intake across the entire first week of life offers a temporal resolution that is rarely available in comparable studies.

Several limitations should be acknowledged. First, the retrospective, single-center design limits the generalizability of our findings and precludes causal inference. Second, reverse causation must be considered: neonates who developed hyperglycemia may have received higher glucose intakes because they were clinically more unstable, rather than the converse. However, several elements argue against this interpretation as the sole explanation. In our NICU, parenteral glucose was prescribed daily according to a standard escalation protocol based on weight and day of life, rather than adjusted upward in response to clinical deterioration; moreover, our exposure variable captures the glucose actually received (not a reactive dose increase) and the multivariable model adjusted for the principal markers of clinical severity (ELBW, neonatal morbidity, perinatal compromise). Although these measures mitigate confounding by indication, they cannot eliminate it entirely. Third, the exposure and the outcome were measured within the same temporal window (the first week of life), which limits our ability to establish the precise temporal sequence between the day the threshold was exceeded and the day hyperglycemia was first detected. The day-by-day analysis showing that glucose intake in cases was already higher than in controls from day 2 provides indirect evidence that increased glucose delivery preceded or coincided with the metabolic complication, but a definitive temporal ordering would require prospective time-to-event analyses. In addition, since the metabolic outcomes were defined as the occurrence of an event during the first week of life, the design does not allow for a precise individual-level assessment of the correspondence between the duration of the exceedance of the threshold and the duration of the associated metabolic complication. Fourth, the threshold of 7 g/kg/day was identified as the data-driven inflection point of the restricted cubic spline analysis performed on the same cohort used in the subsequent multivariable analyses. Although this approach allowed an empirically grounded operationalisation of the exposure, the in-sample derivation of the threshold may have introduced optimism bias and may overestimate the strength of the association compared with what would be observed in an independent cohort. Even after internal validation by non-parametric bootstrap resampling and by repeated stratified 10-fold cross-validation, the strength of the association between exceeding the parenteral dextrose threshold and the metabolic outcomes remains subject to potential optimism bias and may not generalise beyond the present cohort. External validation of the 7 g/kg/day threshold in preterm populations from other centres is therefore required before it can be applied as a definitive risk-stratification cut-off. Fifth, the exclusion of neonates who died or were transferred to another centre within the first 72 h of life may introduce a survivor bias; consequently, the analytic cohort is representative of preterm neonates who survive at least the first three days of life and are not transferred elsewhere in the same window. Sixth, although the local NICU protocol described in Methods Section 2.4 was designed to keep the effective rate of glucose delivery stable across the day, minor unmeasured fluctuations in the instantaneous glucose infusion rate cannot be entirely excluded in a retrospective design. Similarly, although the glucose monitoring schedule was protocol-driven and uniform across the cohort, residual detection bias from minor deviations from the protocolised schedule cannot be entirely excluded. Seventh, 27 neonates (6.9%) were excluded from the multivariable analysis due to missing data on enteral nutrition timing or glycemic outcome classification; however, their baseline characteristics did not differ significantly from the analytic sample, reducing the likelihood of selection bias.

Several directions for future research emerge from the present analysis. First, the 7 g/kg/day threshold requires external validation in a prospective, ideally multicentre cohort of preterm neonates recruited under harmonised parenteral nutrition protocols. Second, a prospective time-to-event design, possibly supported by continuous glucose monitoring to improve the temporal resolution of glycaemic ascertainment, would allow the individual-level sequencing of the exposure-outcome relationship that the present retrospective design cannot establish. Third, complementary preclinical studies in large animal models of prematurity could allow a controlled mechanistic dissection of the relationship between parenteral glucose load, oxidative capacity, and the development of hyperglycaemia and hypertriglyceridaemia, which is not feasible in the clinical setting. Finally, the long-term follow-up of preterm neonates exposed to different parenteral glucose intakes during the early acute phase will be necessary to assess whether the metabolic complications observed in the first week of life translate into neurodevelopmental and growth differences in childhood, in line with our previous work in the area.

## 5. Conclusions

In preterm neonates, exceeding a parenteral dextrose intake of 7 g/kg/day during the first week of life is independently associated with hyperglycemia and hypertriglyceridemia. The day-by-day analysis of glucose intake shows that the divergence between affected and unaffected neonates begins as early as the second day of life, suggesting that the pace of glucose escalation during the early acute phase, not only the absolute intake, plays a role in the development of metabolic complications. As current guidelines define a glucose intake range for this phase but do not specify an optimal escalation trajectory, our findings indicate that, while prospective studies are needed to confirm these observations and to evaluate the impact of targeted glucose escalation strategies on metabolic outcomes in this population, in the meantime a gradual approach to parenteral glucose advancement during the first days of life may be associated with a more favorable metabolic profile.

## Figures and Tables

**Table 1:** Baseline clinical characteristics of the study population.

Variable	Total Population (*n* = 389)
***Prenatal characteristics***	
Maternal age, years	34 (17–52)
Placental abruption, *n* (%)	36 (9.3)
Abnormal Doppler findings, *n* (%)	69 (17.7)
Maternal infectious risk, *n* (%)	136 (35.0)
Antenatal antibiotic prophylaxis, *n* (%)	111 (28.5)
Antenatal steroid administration, *n* (%)	255 (65.6)
Hypertensive disorders, *n* (%)	90 (23.1)
Thyroid disorders, *n* (%)	55 (14.1)
Gestational diabetes, *n* (%)	46 (11.8)
IUGR, *n* (%)	62 (15.9)
***Perinatal characteristics***	
Cesarean section, *n* (%)	336 (86.4)
Cord pH	7.3 (6.8–7.5)
Cord base excess, mEq/L	−5.7 (−27.0 to 8.8)
APGAR score at 5 min	7.6 (1–10)
***Neonatal characteristics***	
Male sex, *n* (%)	211 (54.2)
Gestational age, weeks	29.3 (22–36)
ELBW (≤1000 g), *n* (%)	107 (27.5)
SGA at birth, *n* (%)	68 (17.5)
Birth weight, g	1229 (410–2650)
Birth weight, z-score	−0.35 (−4.0 to 3.1)
Head circumference, cm	27.2 (19–43)
Head circumference, z-score	0.08 (−3.4 to 16.6)
Birth length, cm	37.7 (19–50)

Notes: Data are expressed as mean (range) for continuous variables and *n* (%) for categorical variables. Abbreviations: IUGR, intrauterine growth restriction; ELBW, extremely low birth weight; SGA, small for gestational age.

## Data Availability

The datasets analyzed during the current study are available from the corresponding author on reasonable request due to privacy and ethical restrictions related to sensitive clinical data of a vulnerable population (preterm neonates).

## Supplementary Materials

The following supporting information can be downloaded at: https://www.mdpi.com/article/10.3390/nu18111821/s1. Figure S1: Day-by-day parenteral glucose intake (mean ± 95% CI) during the first week of life in preterm neonates, stratified by primary metabolic outcome (cases vs. controls) for hyperglycaemia (red tones) and hypertriglyceridaemia (orange/green tones). Orange dashed lines indicate the ESPGHAN early-acute recommended limits (5 and 8 g/kg/day, Moltu et al.). The purple dotted line marks the study threshold of 7 g/kg/day. Table S1: Bias-corrected and accelerated (BCa) 95% confidence intervals for the adjusted odds ratios of the multivariable logistic regression models for the two primary metabolic outcomes, obtained by non-parametric bootstrap resampling (1000 stratified resamples, fixed random seed 42). Table S2: Optimism-corrected discrimination and calibration of the multivariable logistic regression models for the two primary metabolic outcomes, obtained by stratified 10-fold cross-validation repeated 100 times (fixed random seed 42).

## References

[1] Van Goudoever J.B., Carnielli V., Darmaun D., Sainz de Pipaon M., ESPGHAN/ESPEN/ESPR/CSPEN working group on pediatric parenteral nutrition (2018). ESPGHAN/ESPEN/ESPR/CSPEN guidelines on pediatric parenteral nutrition: Amino acids. Clin. Nutr..

[2] Embleton N.D., Simmer K., Koletzko B., Poindexter B., Uauy R. (2014). Practice of Parenteral Nutrition in VLBW and ELBW Infants. Nutritional Care of Preterm Infants: Scientific Basis and Practical Guidelines.

[3] Moltu S.J., Bronsky J., Embleton N., Gerasimidis K., Indrio F., Köglmeier J., de Koning B., Lapillonne A., Norsa L., Verduci E. (2021). Nutritional Management of the Critically Ill Neonate. J. Pediatr. Gastroenterol. Nutr..

[4] Stephens B.E., Walden R.V., Gargus R.A., Tucker R., McKinley L., Mance M., Nye J., Vohr B.R. (2009). First-Week Protein and Energy Intakes Are Associated with 18-Month Developmental Outcomes in Extremely Low Birth Weight Infants. Pediatrics.

[5] Joosten K., Verbruggen S. (2022). PN Administration in Critically Ill Children in Different Phases of the Stress Response. Nutrients.

[6] Hay W.W. (2013). Aggressive Nutrition of the Preterm Infant. Curr. Pediatr. Rep..

[7] Sunehag A.L., Haymond M.W., Schanler R.J., Reeds P.J., Bier D.M. (1999). Gluconeogenesis in Very Low Birth Weight Infants Receiving Total Parenteral Nutrition. Diabetes.

[8] Boscarino G., Conti M.G., Gasparini C., Onestà E., Faccioli F., Dito L., Regoli D., Spalice A., Parisi P., Terrin G. (2021). Neonatal Hyperglycemia Related to Parenteral Nutrition Affects Long-Term Neurodevelopment in Preterm Newborn: A Prospective Cohort Study. Nutrients.

[9] Stensvold H.J., Strommen K., Lang A.M., Abrahamsen T.G., Steen E.K., Pripp A.H., Ronnestad A.E. (2015). Early Enhanced Parenteral Nutrition, Hyperglycemia, and Death Among Extremely Low-Birth-Weight Infants. JAMA Pediatr..

[10] Beardsall K., Vanhaesebrouck S., Ogilvy-Stuart A.L., Vanhole C., VanWeissenbruch M., Midgley P., Thio M., Cornette L., Ossuetta I., Palmer C.R. (2013). Validation of the Continuous Glucose Monitoring Sensor in Preterm Infants. Arch. Dis. Child Fetal Neonatal Ed..

[11] Hall N.J., Peters M., Eaton S., Pierro A. (2004). Hyperglycemia Is Associated with Increased Morbidity and Mortality Rates in Neonates with Necrotizing Enterocolitis. J. Pediatr. Surg..

[12] Vitali R., Terrin G., Palone F., Laudadio I., Cucchiara S., Boscarino G., Di Chiara M., Stronati L. (2021). Fecal High-Mobility Group Box 1 as a Marker of Early Stage of Necrotizing Enterocolitis in Preterm Neonates. Front. Pediatr..

[13] Pacella I., Di Chiara M., Prota R., De Luca C., Cardillo A., Potenza E., Grimaldos A.P., Pinna V., Piconese S., Terrin G. (2023). Reduction in Regulatory T Cells in Preterm Newborns Is Associated with Necrotizing Enterocolitis. Pediatr. Res..

[14] Hellerstein M.K. (1999). De Novo Lipogenesis in Humans: Metabolic and Regulatory Aspects. Eur. J. Clin. Nutr..

[15] Vlaardingerbroek H., Vermeulen M.J., Carnielli V.P., Vaz F.M., van den Akker C.H.P., van Goudoever J.B. (2014). Growth and Fatty Acid Profiles of VLBW Infants Receiving a Multicomponent Lipid Emulsion from Birth. J. Pediatr. Gastroenterol. Nutr..

[16] Denne S.C., Kalhan S.C. (1986). Glucose Carbon Recycling and Oxidation in Human Newborns. Am. J. Physiol..

[17] Thureen P.J., Melara D., Fennessey P.V., Hay W.W. (2003). Effect of Low versus High Intravenous Amino Acid Intake on Very Low Birth Weight Infants in the Early Neonatal Period. Pediatr. Res..

[18] Galderisi A., Facchinetti A., Steil G.M., Ortiz-Rubio P., Cavallin F., Tamborlane W.V., Baraldi E., Cobelli C., Trevisanuto D. (2017). Continuous Glucose Monitoring in Very Preterm Infants: A Randomized Controlled Trial. Pediatrics.

[19] Chan B., Lian A., Baer V., Robinson M., Ou Z., Presson A.P., Zinkhan E.K. (2021). An Evaluation to Establish the Acceptable Serum Triglyceride Levels in Neonates Receiving Intravenous Fat Emulsion Infusion in a Multicenter Retrospective Study. Am. J. Perinatol..

[20] Keir A., McPhee A., Wilkinson D. (2014). Beyond the Borderline: Outcomes for Inborn Infants Born at ≤500 Grams. J. Paediatr. Child Health.

[21] Di Chiara M., Laccetta G., Regoli D., Dito L., Spiriti C., De Santis B., Travaglia E., Prota R., Parisi P., Brunelli R. (2023). Delayed Macronutrients’ Target Achievement in Parenteral Nutrition Reduces the Risk of Hyperglycemia in Preterm Newborn: A Randomized Controlled Trial. Nutrients.

[22] Fenton T.R., Cormack B., Goldberg D., Nasser R., Alshaikh B., Eliasziw M., Hay W.W., Hoyos A., Anderson D., Bloomfield F. (2020). “Extrauterine Growth Restriction” and “Postnatal Growth Failure” Are Misnomers for Preterm Infants. J. Perinatol..

[23] Patel A.L., Engstrom J.L., Meier P.P., Kimura R.E. (2005). Accuracy of Methods for Calculating Postnatal Growth Velocity for Extremely Low Birth Weight Infants. Pediatrics.

[24] Harrell F.E. (2015). Regression Modeling Strategies: With Applications to Linear Models, Logistic and Ordinal Regression, and Survival Analysis.

[25] Bonsante F., Iacobelli S., Latorre G., Rigo J., De Felice C., Robillard P.Y., Gouyon J.B. (2013). Initial Amino Acid Intake Influences Phosphorus and Calcium Homeostasis in Preterm Infants—It Is Time to Change the Composition of the Early Parenteral Nutrition. PLoS ONE.

[26] Zamir I., Stoltz Sjöström E., Ahlsson F., Hansen-Pupp I., Serenius F., Domellöf M. (2021). Neonatal Hyperglycaemia Is Associated with Worse Neurodevelopmental Outcomes in Extremely Preterm Infants. Arch. Dis. Child Fetal Neonatal. Ed..

[27] Tottman A.C., Alsweiler J.M., Bloomfield F.H., Gamble G., Jiang Y., Leung M., Poppe T., Thompson B., Wouldes T.A., Harding J.E. (2018). Long-Term Outcomes of Hyperglycemic Preterm Infants Randomized to Tight Glycemic Control. J. Pediatr..

[28] Beardsall K., Vanhaesebrouck S., Ogilvy-Stuart A.L., Vanhole C., Palmer C.R., Ong K., vanWeissenbruch M., Midgley P., Thompson M., Thio M. (2010). Prevalence and Determinants of Hyperglycemia in Very Low Birth Weight Infants: Cohort Analyses of the NIRTURE Study. J. Pediatr..

[29] Embleton N.E., Pang N., Cooke R.J. (2001). Postnatal Malnutrition and Growth Retardation: An Inevitable Consequence of Current Recommendations in Preterm Infants?. Pediatrics.

[30] Ehrenkranz R.A., Dusick A.M., Vohr B.R., Wright L.L., Wrage L.A., Poole W.K. (2006). Growth in the Neonatal Intensive Care Unit Influences Neurodevelopmental and Growth Outcomes of Extremely Low Birth Weight Infants. Pediatrics.

[31] De Nardo M.C., Petrella C., Di Chiara M., Di Mario C., Deli G., Travaglia E., Baldini L., Russo A., Parisi P., Fiore M. (2022). Early Nutritional Intake Influences the Serum Levels of Nerve Growth Factor (NGF) and Brain-Derived Neurotrophic Factor in Preterm Newborns. Front. Neurol..

[32] Mesotten D., Joosten K., van Kempen A., Verbruggen S., ESPGHAN/ESPEN/ESPR/CSPEN working group on pediatric parenteral nutrition (2018). ESPGHAN/ESPEN/ESPR/CSPEN Guidelines on Pediatric Parenteral Nutrition: Carbohydrates. Clin. Nutr..

[33] Derme M., Piccioni M.G., Brunelli R., Crognale A., Denotti M., Ciolli P., Scomparin D., Tarani L., Paparella R., Terrin G. (2023). Oxidative Stress in a Mother Consuming Alcohol during Pregnancy and in Her Newborn: A Case Report. Antioxidants.

[34] Khovidhunkit W., Kim M.-S., Memon R.A., Shigenaga J.K., Moser A.H., Feingold K.R., Grunfeld C. (2004). Effects of Infection and Inflammation on Lipid and Lipoprotein Metabolism: Mechanisms and Consequences to the Host. J. Lipid Res..

