# Supplementary material for: Identifying a Safety Threshold for Parenteral Glucose Intake in the Early Acute Phase of Preterm Neonates
Source: Nutrients. 2026 Jun 5;18(11):1821. doi: 10.3390/nu18111821 (PMC13258789; doi:10.3390/nu18111821)
Supplement: Supplementary file 1 [file nutrients-18-01821-s001.zip › nutrients-4317686-supplementary.pdf]

Supplementary Materials

Manuscript: Identifying a Safety Threshold for Parenteral Glucose Intake in the Early Acute Phase of Preterm Neonates

Internal validation of the multivariable models of Figure 3

Figures

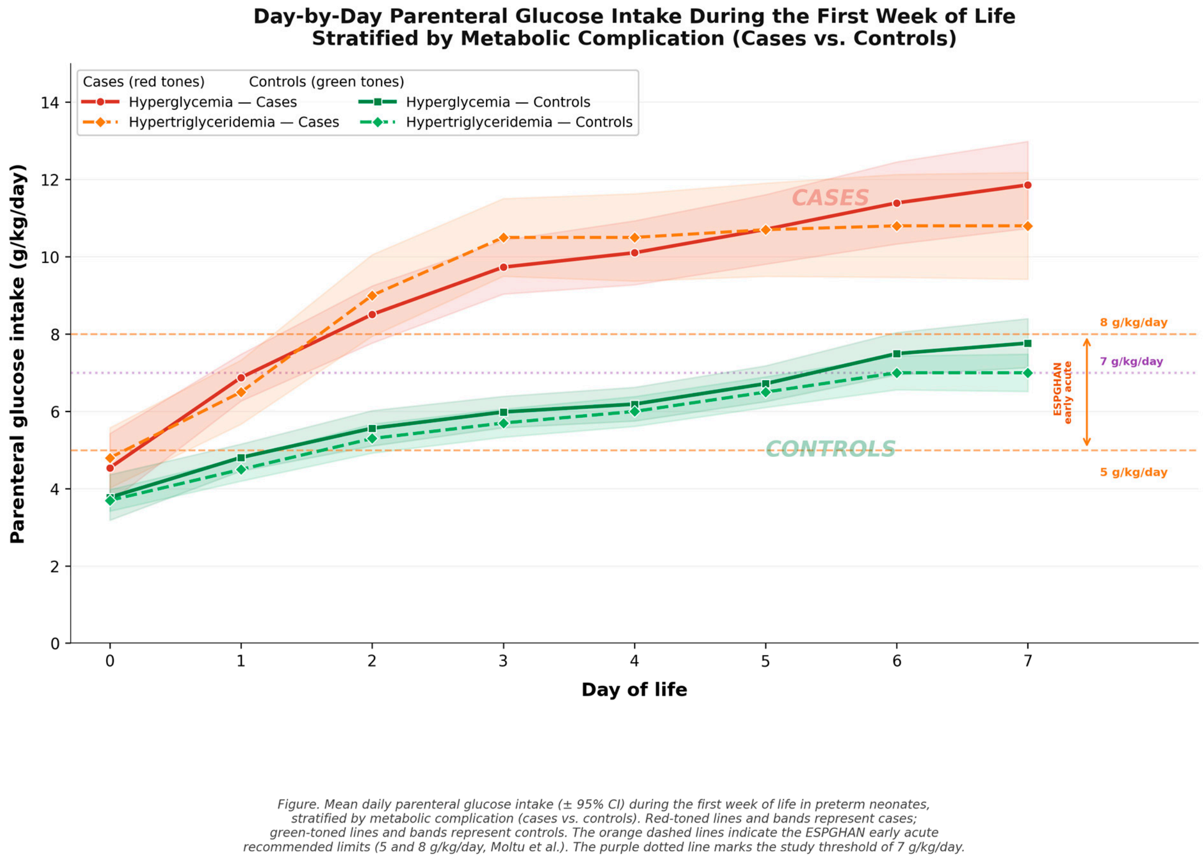

Figure S1. Day-by-day parenteral glucose intake (mean ± 95% CI) during the first week of life in preterm neonates, stratified by primary metabolic outcome (cases vs. controls) for hyperglycaemia (red tones) and hypertriglyceridaemia (orange/green tones). Orange dashed lines indicate the ESPGHAN early-acute recommended limits (5 and 8 g/kg/day, Moltu et al.). The purple dotted line marks the study threshold of 7 g/kg/day.

Table S1. Bias-corrected and accelerated (BCa) 95% confidence intervals for the adjusted odds ratios of the multivariable logistic regression models for the two primary metabolic outcomes, obtained by non-parametric bootstrap resampling (1,000 stratified resamples, fixed random seed 42).

Panel A. Outcome: hyperglycaemia (n = 362 with complete covariate data; 102 events).

Covariate	aOR (apparent)	BCa 95% CI	*p* value
Exceeding glucose target (>7 g/kg/day)	5.76	2.67 to 13.33	< 0.001
ELBW (<1000 g)	2.95	1.53 to 5.35	< 0.001
Gestational age <32 weeks	2.22	0.88 to 6.77	0.098
Delayed enteral nutrition (>72 h)	1.92	0.99 to 3.83	0.053
Neonatal morbidity (sepsis and/or IVH)	1.44	0.84 to 2.79	0.252
Fetal distress (APGAR<5 and/or pH<7.2)	1.25	0.68 to 2.53	0.502

Panel B. Outcome: hypertriglyceridaemia (n = 369 with complete covariate data; 56 events).

Covariate	aOR (apparent)	BCa 95% CI	*p* value
Exceeding glucose target (>7 g/kg/day)	5.23	1.60 to 21.47	0.004
ELBW (<1000 g)	2.67	1.07 to 5.91	0.008
Gestational age <32 weeks	1.07	0.34 to 4.16	0.910
Delayed enteral nutrition (>72 h)	2.09	0.91 to 4.36	0.045
Neonatal morbidity (sepsis and/or IVH)	3.76	1.81 to 7.69	< 0.001
Fetal distress (APGAR<5 and/or pH<7.2)	1.26	0.57 to 2.71	0.537

Notes: aOR, adjusted odds ratio; BCa, bias-corrected and accelerated; CI, confidence interval. The aOR column reports the apparent point estimate from the multivariable logistic regression on the analytic cohort. The BCa 95% CI was computed from 1,000 non-parametric bootstrap resamples stratified by outcome, with the acceleration parameter obtained by leave-one-out jackknife. *p* values are Wald *p* values from the multivariable model on the full sample. Bootstrap fits failed in 0/1,000 resamples for hyperglycaemia and in 8/1,000 resamples for hypertriglyceridaemia (quasi-complete separation in resamples with very few events); BCa intervals were computed on the converged resamples. Analyses performed in Python 3 (statsmodels, scikit-learn, numpy).

Table S2. Optimism-corrected discrimination and calibration of the multivariable logistic regression models for the two primary metabolic outcomes, obtained by stratified 10-fold cross-validation repeated 100 times (fixed random seed 42).

Outcome	C statistic, apparent	C statistic, CV mean	C statistic, CV 2.5 to 97.5 percentile	Optimism (C)	Calibration slope, CV mean	Calibration slope, CV 2.5 to 97.5 percentile
Hyperglycaemia	0.806	0.785	0.776 to 0.791	0.021	0.901	0.858 to 0.937
Hypertriglyceridaemia	0.829	0.796	0.779 to 0.808	0.033	0.861	0.779 to 0.902

Notes: Harrell C statistic, area under the receiver operating characteristic curve. Calibration slope, slope of the logistic regression of the observed outcome on the linear predictor (value of 1 indicates perfect calibration; values below 1 indicate predicted probabilities that are too extreme, consistent with overfitting). CV mean, mean across 100 repetitions of stratified 10-fold cross-validation with random fold assignment. CV 2.5 to 97.5 percentile, empirical range across the 100 repetitions. Optimism (C), apparent minus cross-validated C statistic; positive values indicate optimistic apparent discrimination. The apparent calibration slope is 1 by construction on the development sample and is therefore not reported. Multivariable models retained the original specification of Figure 3.
